# A pharmacovigilance study on patients of bronchial asthma in a teaching hospital

**DOI:** 10.4103/0975-7406.72135

**Published:** 2010

**Authors:** A. N. Jamali, M. Aqil, M. S. Alam, K. K. Pillai, P. Kapur

**Affiliations:** Department of Pharmacy Practice (Pharmacology), New Delhi - 110 062, India; 1 Pharmaceutics, Faculty of Pharmacy, Hamdard University, New Delhi - 110 062, India; 2 Department of Medicine, Majeedia Hospital, Hamdard Nagar, New Delhi - 110 062, India

**Keywords:** Adverse drug reaction, adverse drug reaction monitoring, antiasthmatic, bronchial asthma, pharmacovigilance

## Abstract

**Objective:**

The present study was conducted to monitor adverse drug reactions in patients of bronchial asthma in outpatient department and inpatient department of a university teaching hospital in South Delhi.

**Materials and Methods:**

About 200 patients irrespective of age and sex with established asthma were interviewed during the time period of January 2006 to April 2006 using structured questionnaire. Naranjo’s adverse drug reaction probability scale was used to assess the adverse drug reactions.

**Results:**

A total of 15 adverse drug reactions were reported in 13 out of 200 asthmatic patients. Among the 13 patients reported with adverse drug reactions, 5 (38.5%) were male and 8 (61.5%) patients were female. Maximum percentage of ADRs (2 in 15 prescriptions, 13.3%) observed with montelukast, followed by beclomethasone (1 in 12 prescriptions, 8.3%), salbutamol (6 in 109 prescriptions, 5.5%), and ipratropium (3 in 63 prescriptions, 4.8%).

**Conclusions:**

Montelukast was found to be associated with greater percentage of adverse drug reactions as compared to other antiasthamatics. The above findings are constrained by a small sample size and need to be corroborated by conducting long-term studies using a larger sample size.

Asthma is a disease of airways that is characterized by increased responsiveness of the tracheobronchial tree to a multiplicity of stimuli. It is manifested physiologically by a wide spread narrowing of the passages, which may be relieved spontaneously as a result of therapy, and clinically by paroxysms of dyspnoea, cough and wheezing.[1]

Asthma is one of the most common chronic diseases in the world. It is estimated that around 300 million people in the world currently have asthma.[2]

With the projected increase in the proportion of the world’s population that is urban from 45% to 59% in 2025, there is likely to be a marked increase in the number of asthmatics worldwide over the next two decades. It is estimated that there may be an additional 100 million persons with asthma by 2025.[3] It is estimated that asthma accounts for about 1 in every 250 deaths worldwide. Many of the deaths are preventable, being due to suboptimal long-term medical care and delay in obtaining help during the final attack.[4]

India is projected to become the world’s most populous nation by the year 2050. As a result, further predicted increase in the prevalence of asthma will result in a marked increase in the number of asthmatics.[5] According to National Family Health Survey 2, NFHS-2 (1998-1999) report, the estimated prevalence of asthma in India is 2468 per 100,000 persons. The prevalence was higher in the urban areas (2649 vs. 1966). The prevalence among males was slightly higher (2561). Among those below 15 years of age, asthma was seen in 950 per 100,000 persons. The prevalence rate was 2309 among those in the age group of 15–59 years, while it was 10,375 in those above 60 years of age. The prevalence of asthma in adult males (≥18 years) during 1995–1997 was 3.94% in rural areas. In females of the same age group, the prevalence was 1.27% in urban as well as rural areas. In earlier studies in the 1960s on adults, the prevalence of asthma was 1.89% and 1.76% in Delhi and Patna, respectively.[6]

In a study on students in the age group of 10–18 years in Chandigarh, 2.3% were diagnosed with asthma. The prevalence varied with age and the lowest prevalence was seen among those in the age group of 13–14 years.[7]

World health organization (WHO, 2004) defines “pharmacovigilance” as the science and activities relating to the detection, assessment, understanding, and prevention of adverse drug reactions (ADRs), or any other medicine-related problems.[8]

WHO (1975) defines an ADR as “any response to a drug which is noxious, and unintended, and which occurs at doses normally used in a man for the prophylaxis, diagnosis or therapy of disease, or for modification of physiological function”.[9]

ADR can also be defined as “an appreciably harmful or unpleasant reaction, resulting from intervention related to the use of a medicinal product, which predicts hazard from future administration and warrants prevention or specific treatment, or alteration of the dosage regimen, or withdrawal of the product.”

ADRs constitute a considerable burden of society both financially and in terms of human suffering, and systemized ADR monitoring and reporting may sensitize physicians to rational prescribing.[10] Many reports in the medical literature document the role of pharmacists making specific contribution in reporting ADRs.[11] ADRs that pharmacists report to national pharmacovigilance centers reflect patient concerns about ADRs they experience in relation to the medication they are taking.[12] Also, pharmacists’ reporting of ADRs leads to greater quantity and quality of reporting.[13]

Pharmacovigilance studies for monitoring ADRs related to antiasthmatic agents have been performed by various workers around the globe. Kallergis *et al*. reported significant electrophysiological effects on the atrium, nodes, and ventricle with salbutamol administered by nebulizer. Salbutamol also, significantly decreased the atrial effective refractory period (AERP), and ventricular effective refractory period (VERP).[14] Cushing’s syndrome and its numerous associated complications like diabetes and blood pressure with prolonged unsupervised use of glucocorticoid ocular drops are reported ADRs.[15]

Reports on monitoring of ADRs in India are scarce. A study conducted in Mumbai, India, reports that common adverse effect seen was oral thrush (35%), tremor and palpitation (20%), throat irritation (20%), and cough (10%).[16]

According to Naranjo scale, the probability that the adverse event is related to drug therapy is expressed as definite, probable, possible, or doubtful.[17,18]

A “definite” reaction is one that follows a reasonable temporal sequence after a drug or in which a toxic drug level has been established in body fluids or tissues, followed by a recognized response to the suspected drug, and is confirmed by the improvement on withdrawing the drug and reapper on re-exposure.

A “probable” reaction follows a responsible temporal sequence after a drug, followed by a recognized response to the suspected drug, which is confirmed by withdrawal but not by re-exposure to the drug, and could not be reasonably explained by the known characteristics of the patient’s clinical state.

A “possible” reaction follows a temporal sequence after a drug, possibly followed by a recognized pattern to the suspected drug, and could be explained by characteristic of the patient’s disease.

A reaction is defined as “doubtful” if it is directly related to factors other than a drug.

## Materials and Methods

It was an open and noncomparative study, based on an ADR monitoring form drafted according to WHO monitoring guidelines.[19] The information collected included (age, sex, height, weight), past medical history, present drug treatment, description, assessment, and treatment of ADR. The study was approved by Jamia Hamdard (Hamdard University) Institutional Review Board vide letter number (01/06), dated 12th January, 2006.

The study was carried out in the Medicine Outpatient Department (OPD) and Inpatient Department (IPD) of Majeedia Hospital, a 150-bed teaching hospital situated in Hamdard University campus, New Delhi. ADR monitoring was conducted from January 2006 to April 2006 by a registered pharmacist. A total of 200 patients irrespective of age and sex with established asthma were included in the study. An informed consent was taken from patients participating in the study.

The Naranjo’s probability scale was used for causality assessment of adverse events.[18] They assigned a weighted score to the components used to establish a causal association between drug and adverse events (temporal sequence, pattern of response, withdrawal, re-exposure, alternative, placebo response, drug levels in body fluids or tissues, dose–response relationship, previous patient experience with the drug, and confirmation by objective evidence). These factors were analyzed and scored using the ADR probability scale [Table 1]. Each question could be answered positive (yes), negative (no), or unknown or not applicable (do not know). The ADR was assigned to a probability category from the total score as follows: definite ≥9, probable 5–8, possible 1–4, and doubtful ≤ 0.

**Table 1:** Naranjo’s ADR probability scale

	Yes	No	Do not know Score	Score
Are there previous conclusive reports on this reaction?	+ 1	0	0	
Did the adverse event appear after the suspected drug was administered?	+ 2	−1	0	
Did the adverse event improve when the drug was discontinued or a specific antagonist was administered?	+ 1	0	0	
Did the adverse reaction reappear when the drug was readministered?	+ 2	−1	0	
Are there alternative causes (other than the drug) that could on their own have caused the reaction?	−1	+2	0	
Did the reaction reappear when a placebo was given?	−1	+1	0	
Was the drug detected in the blood (or other fluids) in concentrations known to be toxic?	+1	0	0	
Was the reaction more severe when the dose was increased or less severe when the dose was decreased?	+1	0	0	
Did the patient have the similar reaction to the same or similar drugs in any previous exposure?	+1	0	0	
Was the adverse event confirmed by any objective evidence?	+1	0	0	
			Total Score	

Assessment score: Definite ≥ 9; Probable 5–8; Possible 1–4; doubtful ≤ 0.

## Results

A total of 15 ADRs were reported in 13 out of 200 asthmatic patients. Among the 13 patients reported with ADRs, 5 (38.5%) were male and 8 (61.5%) patients were female. Maximum number of ADRs, 7 (46.7%) were observed in the age group between 41–50 years, followed by 4 (26.7%) in the age range of 31–40 years. Distribution of ADRs among various age groups is given in Table 2.

**Table 2:** ADRs among various age groups of asthmatic patients

Age range	Male (%)	Female (%)	Total (%)
21–30	1 (6.7)	1 (6.7)	2 (13.3)
31–40	1 (6.7)	3 (20)	4 (26.7)
41–50	3 (20)	4 (26.7)	7 (46.7)
51–60	0 (0)	0 (0)	0
61–70	1 (6.7)	1 (6.7)	2 (13.3)
Grand Total			15 (99.9)

Montelukast, a leukotriene antagonist was found to be the commonest drug associated with ADRs (*n*=2, 13.3%), followed by beclomethasone (*n*=1, 8.3%), salbutamol (*n*=6, 5.5%), ipratropium (*n*=3, 4.8%), salmeterol (*n*=2, 4.5%), and fluticasone (*n*=1, 3.2%). The details of ADRs associated with the individual anti-asthmatic drug and their therapeutic classes observed in the study are shown in Table 3. There was no significant difference (*P*>0.05) in ADRs associated with monotherapy and combination therapy [Table 4]. According to the Naranjo’s probability scale, 60% ADRs were found to be possible and 40% as the probable [Table 5]. Highest percentage of ADRs (86.7%) were mild (e.g., nausea, gastrointestinal distress, oral thrush, headache, cough, etc.), which were well tolerated by the patients [Table 6], 13.3% of ADRs were classified as moderate (e.g., sinus tachycardia with salbutamol).

**Table 3:** ADRs, suspected drugs and intervention

Class	Drugs	No. of prescription	No. of ADRs	Adverse reaction	Intervention
β_2_ agonist	Salbutamol	109	2	Tremor	Dechallenged
				Tremor	Dechallenged
			2	Sinus tachycardia	Dechallenged, Digoxin was given
				Sinus tachycardia	Dechallenged, Digoxin was given
			2	Bitter taste, Headache	Symptomatic treatment was given
	Salmeterol	44	1	Bitterness of tongue	None
			1	Cough	Dechallenged, anti-tussive was used
Corticosteroids	Fluticasone	31	1	Oral candidiasis	Counseling was given
	Beclomethasone	12	1	Oral thrush	Counseling was given
Anti cholinergics	Ipratropium	63	1	Dryness of mouth	Dose reduced
			2	Nausea, GI distress	Symptomatic treatment was given (antiemetic, antacid)
Leukotriene antagonist	Montelukast	15	1	Headache	Symptomatic treatment was given (aspirin)
			1	Cough	Symptomatic treatment was given (anti-tussive)
Total		274	15	

**Table 4:** Number of ADRs in patients receiving monotherapy and combination therapy

Therapy	No. of patients	No. of ADRs
Monotherapy	5	7
Combination therapy	8	8
Total	13	15

*P* value>0.05 (not significant)

**Table 5:** Classification of ADRs according to Naranjo’s scale

Assessment score	No. of ADRs	% of ADRs
Doubtful; ≤0	0	0
Possible; 1–4	9	60
Probable; 5–8	6	40
Definite; ≥9	0	0
Total	15	100

**Table 6:** ADR classification on the basis of severity

Severity	No. of ADR	% of ADR
Mild	13	86.7
Moderate	2	13.3
Severe	0	0
Total	15	100

## Discussion

In our study, higher numbers of ADRs were observed in female asthmatic patients as compared to male patients. It might be attributed to females being more sensitive to the effect of drugs as compared to males.[20,21] Majority of the ADRs (86.7%) observed were mild, which were well tolerated by the patients, for example, nausea, gastrointestinal distress, oral thrush, headache, cough, etc.

The number of ADRs was higher with combination therapy as compared to monotherapy although the difference was negligible. In our study, maximum percentage of ADRs was found to be associated with montelukast (*n*=2, 13.3%) that includes headache and cough. Malmstrom *et al* reported worsening asthma 98(25%), headache 68(18%), and upper respiratory tract infection 48(12%) as the most common adverse effects in a pool of 387 asthmatic patients treated with montelukast. They also reported ADRs that mainly included worsening asthma 48(19.1%), headache 47(18.7%), and upper respiratory tract infection 33(13%) out of 251 patients on beclomethasone therapy.[22] Whereas, in a comparative study, Meltzer reported a total of 19 ADRs (7.4%) in 258 asthmatic patients treated with fluticasone with varying distribution of ADRs as follows: oral pharyngeal candidiasis (*n*=8, 3%), hoarseness (*n*=9, 3%), headache (*n*=4, 2%), sore throat (*n*=3, 1%), and insomnia (*n*=3, 1%). They also reported headache in 4 (2%) and sore throat in 1 (<1%) out of 264 patients on montelukast therapy.[23] In the present study, salbutamol was associated with (*n*=6, 5.5%) ADRs(tremor-2, sinus tachycardia-2, bitter taste-1, and headache-1). The administration of salbutamol via inhalational route produced sinus tachycardia as adverse event. The offending drug was withdrawn. The cardiac symptoms declined on dechallenge, but patient was not fully recovered. Hence, digoxin was given as symptomatic treatment. In a foreign study, it was found that the common ADR was tremor (40%), hypokalemia (45.5%), and supraventricular tachycardia (21%); particularly with i.v. infusion, intravenous salbutamol administration.[24] In a study conducted by Bajaj, who reported that most of the ADRs were observed with corticosteroids, followed by β_2_ agonists,[16] it was attributed to the lack of patient counseling on inhaler use.

All drug-related adverse events were evaluated according to the Naranjo’s probability scale [Table 5], 60% of the events were found to be possible ADRs (bitter taste, headache with salbutamol; bitterness of tongue, cough with salmeterol; oral candidiasis with fluticasone; oral thrush with beclomethasone; nausea, GI distress with ipratropium; headache, and cough with montelukast) and 40% as the probable ADRs (tremor, sinus tachycardia with salbutamol; cough with salmeterol; and dryness of mouth with ipratropium). Persistent cough produced on administration of salmeterol was subsided on withdrawing the causative drug. Hence worsening of cough symptoms of asthma were attributed to the above medication.

Most of the drugs were used in the form of inhalation, patients underused their medicine, and short duration of the study could be the reason of lesser number of ADR detection. Other shortcomings of this study are the absence of placebo control group to which we could relate the incidence of ADRs, and the fact that the treatment was known to the physicians and pharmacists.

## Conclusions

The study results highlight the need of ADR monitoring, particularly for use of montelukast and corticosteroids in asthma therapy. It is also prudent to impart proper counseling to the patients regarding the use of different types of inhalers.

## References

[1] Harrison EB, Stephen HL, Dan FL, Dennis KL, Larry JJ (2001). Harrison’s principle of internal medicine.

[2] Masoli M, Fabian D, Holt S, Beasley R (2004). Global burden of asthma. Developed for the Global initiative for asthma.

[3] (2001). World health reports 2001. Fifty facts from the world health report 1998: Global Health Situation and Trends 1955-2025. WHO.

[4] Murray CJ, Lopez AD (1997). Global mortality, disability and the contribution of risk factors: Global burden of disease study. Lancet.

[5] (2005). National commission on Macroeconomics and health- Burden of disease in India.

[6] Jindal SK, Gupta D, Agarwal AN, Jindal RC, Singh V (2000). Study of the prevalence of asthma in adults in north India using a standardized field questionnaire. J Asthma.

[7] Gupta D, Agarwal AN, Kumar R, Jindal SK (2001). Prevalence of bronchial asthma and association with environmental tobacco smoke exposure in adolescent school children in Chandigarh, North India. J Asthma.

[8] (2004). WHO. Pharmacovigilance: Ensuring the safe use of medicines, WHO policy perspective of medicine.

[9] (1975). WHO. Requirements for adverse drug reaction reporting.

[10] Garg KC, Singhal KC, Kumar S (1993). Monitoring the adverse profile of atenolol a collaborative study. Indian J Physiol Pharmacol.

[11] Grootheest KV, Berg LJ, Puijanbrok EP (2004). Do pharmacist reports of adverse drug reactions reflect patient concern?. Pham World Sci.

[12] Groothest KV, Olsson S, Couper M, Berg LJ (2004). Pharmacist role in reporting adverse reaction in an international perspective. Pharmacoepidemiol Drug Saf.

[13] Grootheest KV, Berg LJ, Puijanbrok EP (2002). Contribution of pharmacist to the reporting of ADR. Pharmacoepidemiol Drug Saf.

[14] Kallergis EM, Manios EG, Kanoupakis EM, Schiza SE, Mavrakis HE, Klapsinos NK (2005). Acute electrophysiologic effects of inhaled salbutamol in humans. Chest.

[15] Afandi B, Toumeh MS, Saadi HF (2003). Cushing’s syndrome caused by unsupervised use of ocular glucocorticoids. Endocr Pract.

[16] Bajaj A, Balakrishna S, Sawarkar S (1999). Assessment of therapeutic performances of inhalation aerosols and clinical pharmacist’s services in PFT lab. Indian J Hosp Pham.

[17] Naranjo CA, Pontigo E, Valdenegro C, Gonzalez G, Ruiz I, Busto U (1979). Furosemide-induced adverse reactions in cirrhosis of the liver. Clin Pharmacol Ther.

[18] Naranjo CA, Busto U, Sellers EM, Sandor P, Ruiz I, Roberts EA (1981). A method for estimating the probability of adverse drug reactions. Clin Pharmacol Ther.

[19] Kurakowa T, Correa-Nunes AM, Czarnecki A (2000). Guidelines for setting up and running of pharmacovigilance centre.

[20] Barranco P, Lopez-Serrano MC (1998). General and epidemiological aspects of allergic drug reactions. Clin Exp Allergy.

[21] Riedl MA, Casillas AM (2003). Adverse drug reactions: Types and treatment options. Am Fam Physician.

[22] Malmstrom K, Rodriguez-Gomez G, Guerra J, Villaran C, Piñeiro A, Wei LX (1999). Oral montelukast, inhaled beclomethasone, and placebo for chronic asthma: A randomized, controlled trial. Ann Intern Med.

[23] Meltzer EO, Lockey RF, Friedman BF, Kalberg C, Goode-Sellers S, Srebro S (2002). Efficacy and safety of low-dose fluticasone propionate compared with montelukast for maintenance treatment of persistent asthma. Mayo Clin Proc.

[24] Habashy D, Lam LT, Browne GJ (2003). The administration of beta2-agonists for paediatric asthma and its adverse reaction in Australian and New Zealand emergency departments: a cross-sectional survey. Eur J Emer Med.

